# Epigenetic regulation of metalloproteinases and their inhibitors in rotator cuff tears

**DOI:** 10.1371/journal.pone.0184141

**Published:** 2017-09-13

**Authors:** Mariana Ferreira Leal, Leonardo Caires dos Santos, Adrielle Martins de Oliveira, Paulo Santoro Belangero, Eduardo Antônio Figueiredo, Carina Cohen, Felipe de Seixas Alves, Wânia Hiromi Yanaguizawa, Carlos Vicente Andreoli, Alberto de Castro Pochini, Benno Ejnisman, Marília Cardoso Smith, Maria Teresa de Seixas Alves, Moises Cohen

**Affiliations:** 1 Departamento de Ortopedia e Traumatologia, Universidade Federal de São Paulo, São Paulo, SP, Brazil; 2 Disciplina de Genética, Departamento de Morfologia e Genética, Universidade Federal de São Paulo, São Paulo, SP, Brazil; 3 Departamento de Patologia, Universidade Federal de São Paulo, São Paulo, SP, Brazil; Stellenbosch University Faculty of Medicine and Health Sciences, SOUTH AFRICA

## Abstract

Rotator cuff tear is a common orthopedic condition. Metalloproteinases (MMP) and their inhibitors (TIMP) seem to play a role in the development of joint injuries and in the failure of tissue healing. However, the mechanisms of regulation of gene expression in tendons are still unknown. Epigenetic mechanisms, such as DNA methylation and microRNAs regulation, are involved in the dynamic control of gene expression. Here, the mRNA expression and DNA methylation status of *MMPs* (*MMP1*, *MMP2*, *MMP3*, *MMP9*, *MMP13*, and *MMP14*) and *TIMPs* (*TIMP1-3*) and the expression of miR-29 family members in ruptured supraspinatus tendons were compared with non-injured tendons of individuals without this lesion. Additionally, the gene expression and methylation status at the edge of the ruptured tendon were compared with macroscopically non-injured rotator cuff tendon samples from the anterior and posterior regions of patients with tendon tears. Moreover, the possible associations between the molecular alterations and the clinical and histologic characteristics were investigated. Dysregulated expression and DNA methylation of *MMP* and *TIMP* genes were found across the rotator cuff tendon samples of patients with supraspinatus tears. These alterations were influenced at least in part by age at surgery, sex, smoking habit, tear size, and duration of symptoms. Alterations in the studied *MMP* and *TIMP* genes may contribute to the presence of microcysts, fissures, necrosis, and neovascularization in tendons and may thus be involved in the tendon healing process. In conclusion, MMPs and their inhibitors are regulated by epigenetic modifications and may play a role in rotator cuff tears.

## Introduction

Rotator cuff degeneration is a very common orthopedic condition, and there are multiple factors that eventually lead to a full-thickness rotator cuff tear [1]. The incidence rate of degenerative rotator cuff tears increases with age; thus, such tears can become an increasingly prevalent clinical problem [2]. Surgical repair of tendon tears significantly improves pain and function; however, retearing of the rotator cuff is not an infrequent occurrence [2].

Several studies have investigated the molecular alterations involved in tendon tears and in the failure of cuff healing (for a review, see [1–4]). An improved understanding of the regulation of gene expression in normal and injured tendons is important toward guidance in patient management and the development of new therapeutic options.

A normal tendon mainly consists of collagen fibrils [5]. Schirachi et al. [6] showed that the expression of both type I and type III collagen increases in the ruptured tendon of rotator cuff. Additionally, our group also found increased mRNA expression of *COL1A1* and *COL3A1* collagens [7]. Increased expression of some extracellular matrix (ECM) proteins, such as fibronectin and tenascin, in injured tendon has been reported previously [3, 8, 9]. Therefore, the maintenance of the ECM is crucial to the tendon resistance to mechanical forces and the repair response after injury [3].

Matrix metalloproteinases (MMPs) are a large group of proteolytic enzymes responsible for the tissue remodeling and degradation of the ECM [10]. These enzymes are classified based on their substrate preference into collagenases (e.g., MMP1 and MMP13), stromelysins (e.g., MMP3), and gelatinases (e.g., MMP2 and MMP9). An additional transmembrane anchor domain can be found in the membrane-type MMPs (MT-MMPs) [10]. These proteins are inhibited by tissue inhibitor of metalloproteinases (TIMP), including TIMP1-3 [10]. Balance between MMPs and TIMPs is necessary for tissue maintenance and remodeling.

The altered MMP and TIMP expression could contribute to the etiology of tendon disorders (see review [11]). Nevertheless, few studies have compared injured supraspinatus tendon samples of individuals with rotator cuff tears with non-injured tendon samples [12–17], and only a small number of those have applied a quantitative approach to evaluate the gene expression [17]. Thus, the role of MMP and TIMP expression in rotator cuff tears still needs to be further investigated. Moreover, the epigenetic mechanisms involved in the regulation of their expression in tendons have not been evaluated.

DNA methylation is the most widely studied epigenetic modification. The addition of a methyl group to a cytosine nucleotide results in the presence of 5-methylcytosine (5-mC). Methylated cytosines are more frequently detected in the context of CpG dinucleotides, which are clustered in regions called CpG islands [18]. The presence of methylated CpG sites or CpG islands can contribute to the gene silencing [18] and potentially to translational repression. Although reversible, alterations in DNA methylation may have an effect on the structure and homeostasis of tendons.

MicroRNAs (miRNAs) are short endogenous nonprotein coding RNAs that mediate the posttranscriptional regulation by binding to the 3’ untranslated region (3’ UTR) of target mRNAs, leading to translational inhibition or mRNA degradation. miRNAs can be master regulators of gene expression and influence cell activities and events [19]. As other epigenetic modifications (including DNA methylation), dysregulation of miRNA expression may contribute to modifications at tissue structure and function. The human miR-29 family consists of six miRNAs: *miR-29a-3p*, *miR-29a-5p*, *miR-29b-3p*, *miR-29b-5p*, *miR-29c-3p*, and *miR-29c-5p*. Among the many targets of miR-29 are multiple collagens, integrins, and several metalloproteases [20]. The dysregulated expression of a miR-29 family member may have a role in rotator cuff tears.

The present study aimed to compare the mRNA expression and DNA methylation status of *MMP* and *TIMP* genes and the expression of *miR-29* family members at the edge of the ruptured supraspinatus tendon with the control tendon of individuals without rotator cuff injury. Additionally, the gene expression and methylation status at the edge of the supraspinatus ruptured tendon were compared with macroscopically non-injured rotator cuff tendon samples from the same patients. Moreover, the possible associations between the molecular targets in tendon samples and the clinical and histologic characteristics were investigated.

## Material and methods

The study was approved by the ethics committee of Universidade Federal de São Paulo (UNIFESP; approval number: 1918/11) and all individuals signed a written informed consent before data and sample collection.

### Patients

Tissue samples were obtained from 40 patients undergoing arthroscopic rotator cuff repair at the São Paulo Hospital of the UNIFESP, Brazil. The following inclusion criteria were applied: age between 30 and 70 years old, presence of degenerative full-thickness supraspinatus tears diagnosed by physical (impact tests, Jobe’s test, supra and infraspinatusus tests and Patte’s test) and image (magnetic resonance imaging; MRI) examination and confirmed in surgery, at least 6 months of conservative treatment, and no corticosteroid use (oral or infiltration) within 3 months. All patients presented a subscapular tendon without ruptures as detected by physical examination (Gerber’s lift off test), MRI and arthroscopic examination. The arthroscopic procedure was done in beach chair position and biopsies were done by posterior, anterior and lateral portals. Patients with prior shoulder or orthopedic surgery or a history of glenohumeral arthritis and labrum pathology were excluded from the study. Patients with traumatic tears, tears greater than 5 cm [massive tears according to Cofield [21]], or fatty infiltration greater than grade 2 according to Fuchs et al. [22] were also excluded.

Additionally, 11 patients operated on for two-part proximal humeral fractures (fractures located at the surgical neck [23]) composed a control group. These patients did not complain of shoulder pain before trauma, or present a history of rotator cuff tears or any clinical or radiologic (X-ray and ultrasound) indications of this condition. During the surgical procedure, no macroscopic supraspinatus tendon degeneration or tear was observed. All control patients were physically active.

Table 1 shows the main clinical outcomes of the studied cases and controls (for more details, see table S2 File).

**Table 1:** Distribution of the clinical and histological variables of rotator cuff tear patients and controls.

Variables	Cases	Controls	p-value
Age at surgery, years (mean ± SD)	56.2±11.1	57.5±14.1	0.75^a^
Sex (% male)	47.5%	45.5%	0.59^b^
Smoking habit (% smokers)	12.8%	22.2%	0.39^b^
Age at onset, years (mean ± SD)	55.6±10.5	-	-
Duration of condition, months (mean ± SD)	14.5±17.6	-	-
Tear size, cm (mean ± SD)	2.5±1.1	-	-
Tear size (% small to medium)	74.4%	-	-
Fissure (%)	11.5%	-	-
Necrosis (%)	15.4%	-	-
Myxoid degeneration (%)	88.5%	-	-
Microcysts (%)	38.5%	-	-
Dystrophic calcification (%)	0%	-	-
Neovascularization (%)	23.1%	-	-

SD: standard deviation.

^a^p-value by t-test for independent samples

^b^p-value by Chi-square test.

### Tissue samples

Injured and non-injured specimens about 2 mm^3^ in size were obtained from tendons as previously described [7]. From the patients, tissue samples representative of the three sectors of the rotator cuff according to Habermeyer et al. [24], namely, the central cuff (CC), posterior cuff (PC), and anterior cuff (AC), were biopsied (S1 Fig). The CC samples (the torn supraspinatus edge) represented the macroscopically injured supraspinatus tendon, and the most degenerated site of the tear was chosen for this biopsy in arthroscopic view from posterior portal. The PC samples represented a rotator cuff tendon without macroscopic alteration and with native footprint insertion. The AC samples represented the subscapular tendon, a macroscopically non-injured tendon of the rotator cuff.

A supraspinatus sample was also obtained from the controls (central cuff of external controls; EC) during open surgery for traumatic acute proximal humeral fractures.

All tissue specimens were immediately immersed in Allprotect Tissue Reagent (Qiagen, USA) and stored at -20°C until nucleic acid extraction. In the CC region of 26 (65%) patients, a second tissue sample was obtained, which was formalin-fixed and paraffin-embedded (FFPE) for the histologic evaluation.

### Histology

The FFPE tissue sections were stained with hematoxylin and eosin. The CC samples were classified according to the presence or absence of fissures, necrosis, myxoid degeneration, microcysts, dystrophic calcification, and neovascularization.

### DNA/RNA/miRNA extraction

Total DNA and RNA was extracted from 10–20 mg of tissue sample using an AllPrep DNA/RNA/miRNA Mini Kit (Qiagen, USA). DNA and RNA concentration and quality were immediately determined using a Nanodrop ND-1000 (Thermo Scientifc, USA) and the integrity of the RNA was verified by gel electrophoresis on a 1% agarose gel. Aliquots of DNA and of the total RNA were stored at -80°C until further use.

### mRNA and microRNA expression analysis

Gene expression was evaluated by reverse-transcription quantitative polymerase chain reaction (RT-qPCR). RT-qPCR gene expression quantifications were performed according to MIQE guidelines [25].

For mRNA expression analysis, cDNA was synthesized from 300 ng of RNA using a High-Capacity cDNA Reverse Transcription Kit (Life Technologies, USA). To determine expression of the studied genes, reactions were performed with the 300 ng of cDNA input using TaqMan Low-Density Array (TLDA) cards (Life Technologies, USA) and ViiA 7 Real-Time PCR System (Life Technologies, USA). The *HPRT1*, *TBP* and *ACTB* genes were selected as internal controls to standardize the sample input amount [7]. All qRT-PCR reactions were performed in triplicate for all target and reference genes (S1 Table).

For miRNA expression analysis, cDNA was synthesized from 100 ng of total RNA using an oligonucleotide pool for each evaluated gene using TaqMan^®^ MicroRNA Reverse Transcription Kit (Life Technologies, USA). We performed the pre-amplification step as suggested by the manufacturer and the expression of the studied miRNA was performed in quadruplicate reactions using TaqMan^®^ inventoried Assays-on-Demand probes (Life Technologies, USA) and ViiA 7 Real-Time PCR System (Life Technologies, USA). *U6* gene was selected as internal control to standardize the sample input amount. For each sample, the target and reference genes (S1 Table) were assayed on the same plate to exclude technical variations.

The expression of target genes across the samples was calculated using the equation ΔCrt (relative threshold), in which [ΔCrt = target gene Crt–the mean of reference genes Crt]. A lower cycle threshold value (Crt) indicates higher gene expression.

### DNA methylation analysis

The methylation pattern and frequency of *MMP* and *TIMP* genes were evaluated by next-generation sequencing. The EZ DNA Methylation-Lightning kit (Zymo Research, USA) was used to modify the gDNA (400 ng) by applying bisulfite treatment, converting unmethylated cytosines into uracils, and leaving methylated cytosines unchanged. All DNA bisulfite conversion reactions were done in duplicate.

The specific primers used for the gene promoters are described in S2 Table. The primers used for the *MMP13* methylation status analysis have been previously described [26, 27]. The other primers were designed by using the software Methprimer [28] or Methyl Primer Express^®^ version 1.0 (ThermoFisher, USA).

Two equimolar pools of amplicons were generated per sample and about 10–50 ng of DNA was used as the input for library preparation. Sequencing was done on a PGM sequencer (Ion Torrent; Thermo Fisher, USA) with the use of the Ion PGM^TM^ Hi-Q^TM^ Sequencing kit (Thermo Fisher, USA) and the Ion 318 Chip kit v2 (Thermo Fisher, USA). S1 File presents further information about targeted bisulfite amplicon sequencing.

Bisulfite-modified DNA reads were aligned to the amplicon reference sequences by using Bismark version 0.16.3 [29] with Bowtie version 2.2.9. Only reads longer than 50 bp were used for subsequent methylation extraction, and only one mismatch was tolerated. The percentage methylation of cytosines in CpG, CHG or CHH context (where H can be either A, T or C) was calculated individually for each context following the equation:

%methylation(context)=100*methylatedCs(context)/(methylatedCs(context)+unmethylatedCs(context)).

Non-CpG methylation was used as an internal upper-bound estimate of the inefficiency of bisulfite conversion. All samples presented less than 1% methylation in a CHG (mean, 0.3%) and a CHH (mean, 0.4%) context.

To quantify the DNA methylation in a given set of genomic regions, the mean DNA methylation levels across CpG sites for each region were first calculated. Then, the mean of the means for the whole region set was obtained. Small- and low-coverage region sets were filtered out to reduce background noise [30]. A minimum of 100 CpG measurements across samples was required for each region set; as a result, two CpG sites of *MMP9* were not included in the analyses (S2 Table).

### Statistical analysis

We used general linear model (GLM) test to compare the mRNA expression between case (CC samples) and control samples (EC), as well as clinical and histological variables. Since EC samples do not represent the same portion of the cuff as AC and PC samples, we did not directly compare these groups. GLM for repeated measures followed by Bonferroni post-hoc test was performed to compare the gene expression among paired tendon samples of rotator cuff patients (CC, PC and AC samples).

In addition, t-test for independent samples was used to compare the age between cases and controls. These data are shown as the mean ± standard deviation (SD).

miRNA expression and DNA methylation (most of CpG sites) did not present a normal distribution. Therefore, Mann-Whitney test was used to compare the miRNA expression or DNA methylation between case and control samples, as well as clinical and histological variables. Wilcoxon analysis followed by Bonferroni corrections was performed to compare the miRNA expression or DNA methylation among paired tendon samples of rotator cuff patients. These data are shown as the median and interquartile range (IQR).

Pearson’s or Spearman (non-parametric) correlation was applied to evaluate the possible correlation between quantitative variables, such as expression data, percentage of DNA methylation, age at surgery or duration of symptoms.

Chi-square test was carried out to compare the sex, smoking status and type of job distribution between cases and controls. Chi-square test was also used to compare histological and clinical variables.

A p-value of < 0.05 was considered statistically significant for the analysis. The alpha was adjusted when necessary (Bonferroni corrections).

## Results and discussion

S2 File presents individual data concerning gene expression and DNA methylation of cases and controls.

### mRNA expression in tendon samples

*MMP13* expression was relatively low in the tendon samples and was detected in only 79 tissue samples from 32 cases and 7 samples from controls.

**Fig 1:**
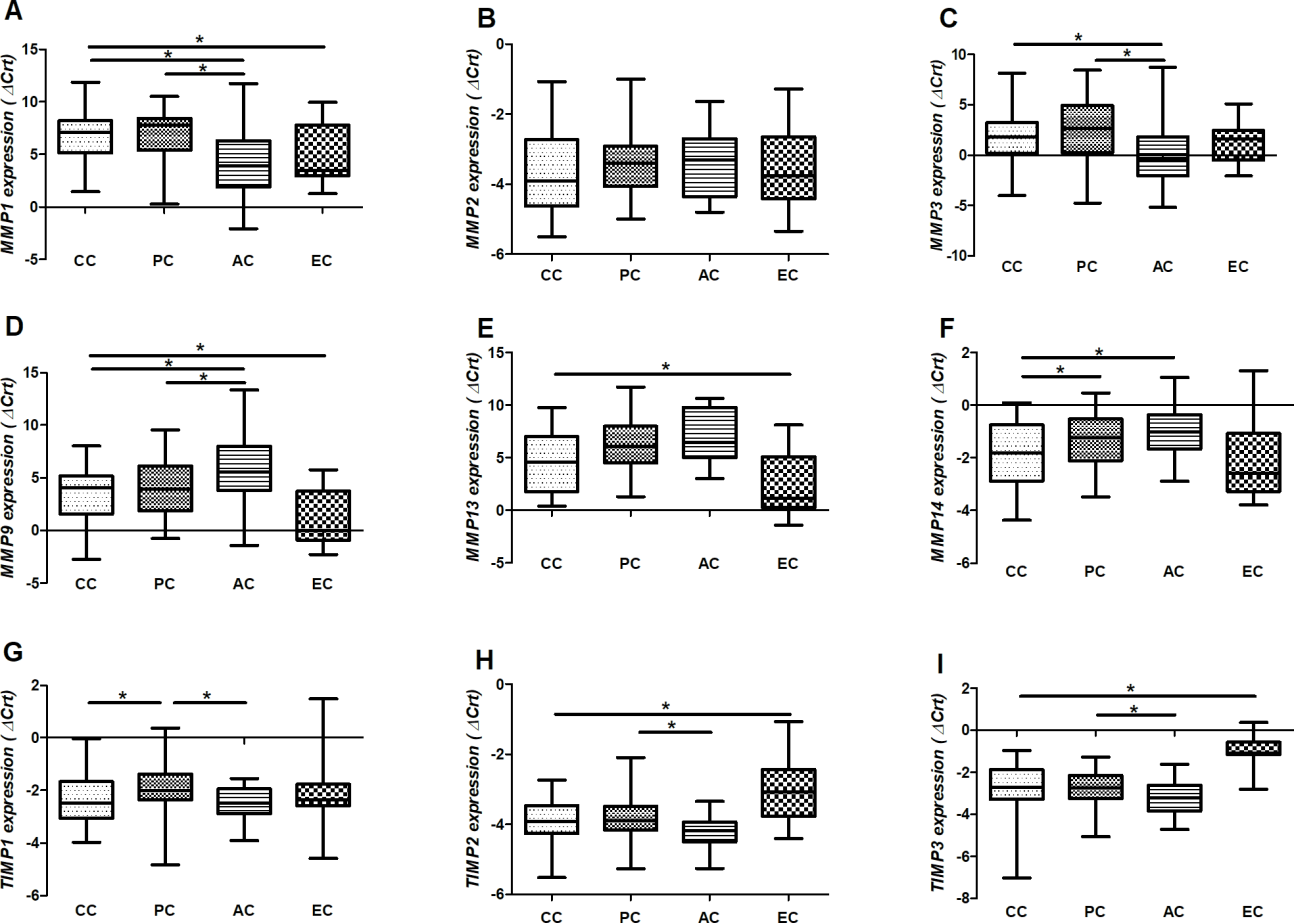
*MMP* and *TIMP* expression in tendon samples of individuals with and without rotator cuff tears. A) *MMP1*; B) *MMP2*; C) *MMP3*; D) *MMP9*; E) *MMP13*; F) *MMP14*; G) *TIMP1*; H) *TIMP2*; I) *TIMP3*. A lower delta cycle threshold value (ΔCrt) indicates higher gene expression. CC: central cuff (the torn supraspinatus edge); PC: posterior cuff, which represents a supraspinatus tendon sample without macroscopic alteration with native footprint insertion; AC: anterior cuff (subscapular tendon); EC: external control representing tendon samples of patients without rotator cuff tears. *Significant difference between groups (p<0.05) by GLM test for the comparisons between cases and controls or by GLM for repeated measures followed by Bonferroni post-hoc test for the comparisons involving multiple samples of patients with rotator cuff tears.

Decreased expression of *MMP1* (p = 0.049; Fig 1A), *MMP9* (p = 0.008; Fig 1D), and *MMP13* (p = 0.043; Fig 1E) was found in the supraspinatus tendon samples of cases compared with controls. Conversely, the expression of *TIMP2* (p = 0.001; Fig 1H) and *TIMP3* (p<0.001; Fig 1) were increased in the tendon tear samples compared with controls.

*MMP1*, *MMP3*, *MMP9*, *MMP14*, *TIMP1*, *TIMP2* and *TIMP3* expression differed among the tendon samples of individuals with rotator cuff tears (p<0.05 for all comparisons, Fig 1). The CC samples showed increased *MMP14* (p = 0.028) and *TIMP1* (p = 0.025) expression compared with the PC samples. Compared with the AC samples, the CC samples had decreased *MMP1* (p<0.001) and *MMP3* (p = 0.009) and increased *MMP9* (p = 0.001) and *MMP14* (p<0.001) expression. In the PC samples, the expression of *MMP1* (p = 0.002), *MMP3* (p = 0.002), *TIMP1* (p = 0.003), *TIMP2* (p = 0.008), and *TIMP3* (p = 0.031) were decreased compared with the AC samples. Conversely, the *MMP9* (p = 0.045) expression was increased in the PC samples compared with the AC samples. The comparison of the *MMP13* expression among the tissue specimens of patients with rotator cuff tears included only 12 matched samples from each tendon region.

Riley et al. [14] previously showed decreased MMP2/MMP9/MMP13 and MMP3 activity in ruptured supraspinatus tendons. However, few studies have investigated the mRNA expression of *MMPs* and *TIMPs* in tendon samples of patients with rotator cuff tears, especially evaluating the results using suitable reference genes [7]. Chaudhury et al. [17], by applying a large-scale approach, described that torn rotator cuff tendons presented increased *MMP13* and decreased *MMP3* expression in small/medium tears; however, *MMP13* expression was not further demonstrated. Our findings regarding *MMP3* expression agree in part with those of Chaudhury et al. [17]. Both MMP3 and MMP13 degrade collagens, fibronectin, and tenascin [31], and their targets seem to present increased expression in rotator cuff tendon tears [11].

Shindle et al. [32] described higher *MMP9* and *MMP13* expression in the injured supraspinatus tendon of patients with full-thickness tears compared with patients with partial tears. However, similarly to the present study, the authors applied the delta Ct (ΔCt) method. A higher ΔCt indicates decreased expression because samples with low copies of an mRNA molecule present later amplification. Thus, Shindle et al. [32] found decreased expression of *MMP9* and *MMP13* in full-thickness tears, which is supported by our findings. It is important to note that this study evaluated only full-thickness tears. At the transcriptional level, our results suggest that there is an attempt to repair the tissue structure at the edge of the ruptured supraspinatus; however, the clinical observations indicate that increased expression of ECM genes, together with decreased expression of MMPs and increased expression of TIMPs, is not enough to repair this tissue, at least in patients with full-thickness tears.

It is important to note that the present study found a significant decrease of *MMP9* expression in injured tendons compared with EC samples; the expression, however, was increased compared with AC samples. The subscapularis tendon is functionally and organizationally distinct from the supraspinatus and thus responds to mechanical loading in a different manner, which may alter the gene expression profile [33]. Therefore, this tendon may not be a perfect control for the study of supraspinatus tendon tears. On the other hand, Reuther et al. [34] demonstrated that injuries in supraspinatus and infraspinatus of rats may lead to molecular and histologic alterations in non-injured tendons (as the subscapular), as well as in other shoulder joint tissues. Moreover, the subscapular from patients with full-thickness rotator cuff tears has been suggested as a useful model of early human tendon injury [33, 35, 36]. Thus, the use of matched supraspinatus and subscapular specimens may help in understanding the dynamic regulation of the gene expression during the degenerative process in tendon samples.

In an experimental murine model of tendon repair, *Mmp9* expression was shown to be increased during the early inflammatory period of healing, followed by a rapid decrease [37]. In this same model, the *Mmp14* expression was involved in the transition from fibroblastic granulation tissue to a more organized collagen structure. Therefore, the increased expression of *MMP9*, *MMP13* (not significant), and *MMP14* in CC compared with AC samples suggest that the edge of the torn supraspinatus has an “active lesion” presenting an inflammatory as well as degenerative process.

It is important to highlight that few significant differences were observed between CC and PC samples, suggesting that the molecular alterations in the supraspinatus tendon are not restricted to its edges.

### miR-29 expression in tendon samples

Dysregulated expression of *MMPs* and *TIMPs* has been associated with rotator cuff tears; thus, we investigated epigenetic factors that may contribute to the regulation of such expression. We investigated whether the expression of miR-29 family members was inversely correlated with the mRNA level of the studied protein-coding genes because it may indicate an involvement in the regulation of gene expression.

**Fig 2:**
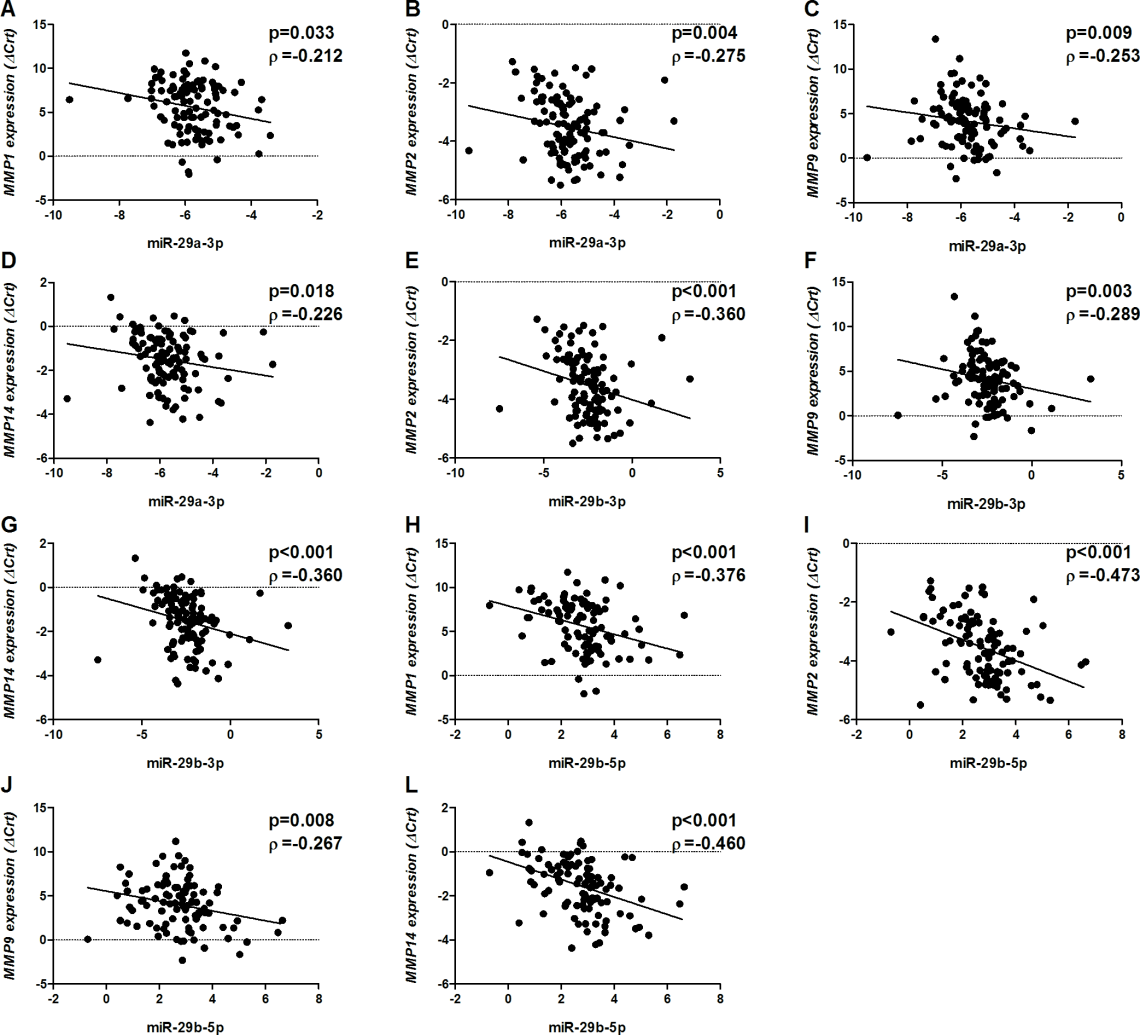
Inversely correlation between miRNAs and MMPs. *miR-29a-3p* was significantly correlated with *MMP1* (A), *MMP2* (B), *MMP9* (C) and *MMP14* (D); *miR-29b-3p* was significantly correlated with *MMP2* (E), *MMP9* (F) and *MMP14* (G); *miR-29b-5p* was significantly correlated with *MMP1* (H), *MMP2* (I), *MMP9* (J) and *MMP14* (L).

The expression of *miR-29c-3p* and *miR-29c-5p* in the tendon samples was not detected with the inputs used in the present study. *miR-29a-3p*, *miR-29b-3p* and *miR-29b-5p* were found to be inversely correlated with *MMP2*, *MMP9* and *MMP14* (p<0.05 for all comparisons, Fig 2). *miR-29a-3p* and *miR-29b-5p* were also inversely correlated with *MMP1* (p<0.05 for all comparisons, Fig 2). These findings agreed with miRTarBase and TargetScan databases. Although these miRNAs may in part be responsible for the posttranscriptional regulation of *MMPs* in the tendon samples, the expression of the studied miR-29 family members did not differ between injured and non-injured tendons (p>0.05 for all comparisons; S2 Fig). Thus, these miRNAs do not seem to be the main contributing factors regulating the ECM genes studied here.

### DNA methylation is involved in the gene expression regulation in tendons

**Fig 3:**
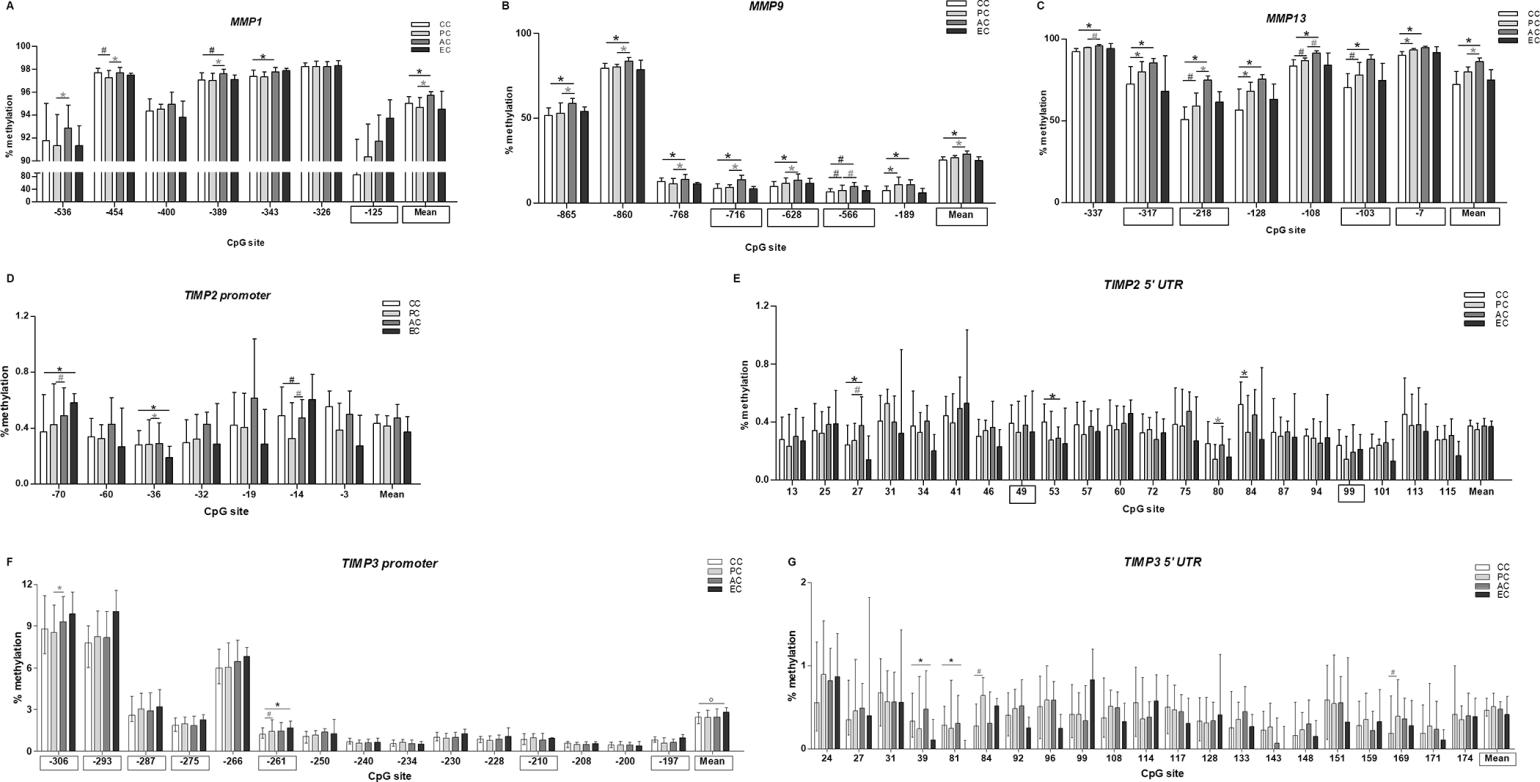
*MMP* and *TIMP* methylation in tendon samples of individuals with and without rotator cuff tears. A) *MMP1* promoter; B) *MMP9* promoter; C) *MMP13* promoter; D) *TIMP2* promoter; E) *TIMP1* 5’UTR; F) *TIMP3* promoter; G) *TIMP3* 5’UTR. CC: central cuff (the torn supraspinatus edge); PC: posterior cuff, which represents a supraspinatus tendon sample without macroscopic alteration with native footprint insertion; AC: anterior cuff (subscapular tendon); EC: external control representing tendon samples of patients without rotator cuff tears. *Significant difference between groups, in which p<0.05 for the comparison between CC and EC samples (Mann-Whitney test) and p<0.016 for the comparisons involving multiple samples of patients with rotator cuff tears (Wilcoxon followed by Bonferroni post-hoc test). ^#^0.0167<p-value<0.05 by Wilcoxon test. Rectangles highlight the CpG sites in which an inverse correlation between DNA methylation frequency and gene expression was detected.

The genes that presented a differential expression between CC and EC were selected for the DNA methylation analysis; these were *MMP1*, *MMP9*, *MMP13*, *TIMP2*, and *TIMP3* (Fig 3). These genes contain CpG islands according to the Genome Browser (h19) or Methprimer software. The DNA methylation of each gene was evaluated only in samples with corresponding mRNA expression data.

The gene expression (a lower ΔCrt indicates increased expression) was inversely correlated with the methylation at the promoters of *MMP1* (CpG -125), *MMP9* (CpGs -716, -628 and -566), *MMP13* (CpGs -7, -103, -218 and -317), and *TIMP3* (CpGs -306, -293, -287, -275, -261, CpG -210 and CpG -197) (p<0.05 for all comparisons; S3 Fig). The mean DNA methylation level at the promoter of *MMP1*, *MMP9*, *MMP13* and *TIMP3* was also inversely correlated with their gene expression (p<0.05 for all comparisons; S3 Fig). The mean DNA methylation level at the 5’UTR of *TIMP3* was also inversely correlated with the gene expression (p = 0.031, ρ = 0.197; S3 Fig; Spearman correlation coefficient). A low frequency of DNA methylation was detected at the promoter (Fig 3D) and 5’ UTR of *TIMP2 *(Fig 3E). Albeit for the low methylation level, the *TIMP2* expression was inversely correlated with the DNA methylation at only 2 of the 28 CpG sites studied: CpG +49 and +99, both of which were at the 5’UTR (p<0.05 for all comparisons; S3 Fig). Our data implicated a role of DNA methylation in the regulation of *MMP* and *TIMP* expression in tendons. It is important to highlight that the CpG sites -125 of *MMP1* and -716 and -566 of *MMP9* have been previously annotated in the Encyclopedia of DNA Elements (ENCODE) based on analysis of other tissues.

We also identified significant differences in the DNA methylation of *MMP1*, *MMP9*, *MMP13*, *TIMP2* and *TIMP3* gene between tissue samples from injured and non-injured rotator cuffs. Between cases and controls, the DNA methylation frequency differed only at the CpG sites -36 (p = 0.031) and -70 (p = 0.012) of *TIMP2* (Fig 3D) and at -261 (p = 0.025), +39 (p = 0.049), and +81 (p = 0.020) of *TIMP3* (Fig 3E and 3F). The mean methylation level in the *TIMP3* promoter region tended to be decreased in cases compared with controls (p = 0.054; Fig 3E).

The DNA methylation frequency at several CpG sites of *MMP1*, *MMP9*, and *MMP13* was significantly different among the tendon regions of patients with rotator cuff tears (p<0.05; Fig 3A–3C). The mean DNA methylation level of *MMP1*, *MMP9*, and *MMP13* differed significantly between the CC and the AC samples (p = 0.001, p<0.001, and p<0.001, respectively) and between the PC and the AC samples (p = 0.002, p<0.001, and p = 0.007, respectively). However, considering the correlation between methylation and expression, it was surprising that the DNA methylation of the *MMP1* gene in the AC samples was increased. The frequency at a few CpG sites of *TIMP2* and *TIMP3* significantly differed among the tendon samples of patients with rotator cuff tears (Fig 3D–3G).

Few significant differences in the DNA methylation frequency involving mainly *TIMP2* and *TIMP3* were observed between the CC and the EC samples. These findings support that these *TIMPs* have a role in tendon tears. However, the identification of other CpG sites that were differentially methylated between cases and controls was limited by the small number of control samples and the large variation among the specimens.

Although the DNA methylation of *MMP1* was inversely correlated with the gene expression, the AC samples (which presented increased *MMP1* expression compared with the CC and PC samples) showed increased methylation at some CpG sites. Considering the subscapularis from patients with full-thickness rotator cuff tears as a model of early human tendon injury [33–36], this finding suggests that the *MMP1* methylation precedes the downregulation of *MMP1* expression, which is also regulated by other transcriptional and epigenetic factors.

Increased methylation at several CpG sites of *MMP9* and *MMP13* was found in the AC samples compared with the CC and PC samples; this agrees with the gene expression findings and highlights the dynamic regulation of these genes in the tendon samples.

It is worth to note that correction for multiple testing (multiple CpG sites) was not carried out in the analysis of the DNA methylation data. Because no similar study has been published previously, we chose to reject the null hypothesis to prioritize the biological data that may be true (type I error) rather than deny any important molecular event involved in the rotator cuff tear due to statistical rigor (Type II error). This study is the first to investigate the possible involvement of epigenetic modifications in the etiology of rotator cuff tears.

### Gene expression and methylation in tendons influenced by clinical outcomes

**Fig 4:**
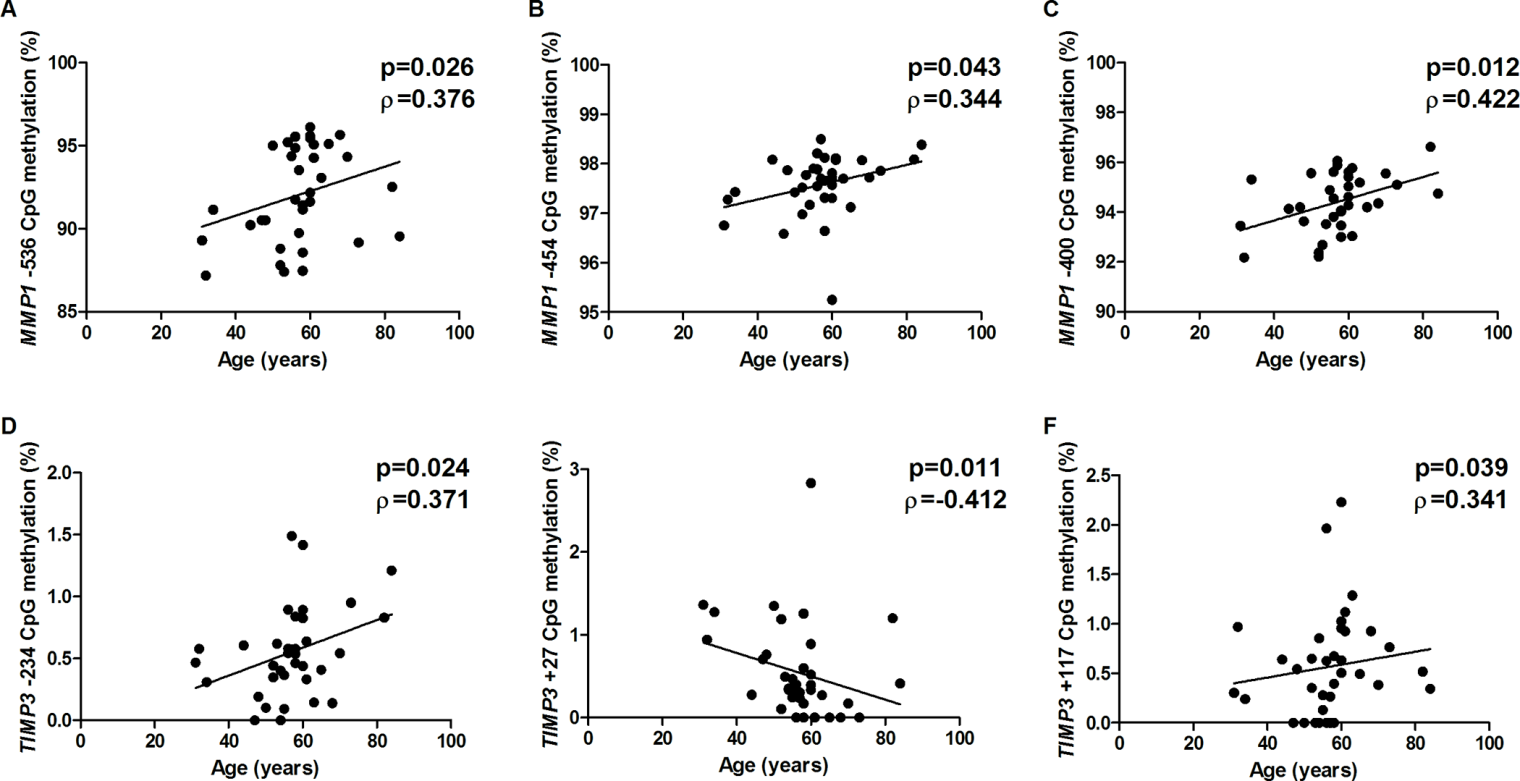
Correlation between DNA methylation and age at surgery in rotator cuff tears. A) CpG site -536 of *MMP1*; B) CpG site -454 of *MMP1*; C) CpG site -400 of *MMP1*; D) CpG site -234 of *TIMP3*; E) CpG site +27 of *TIMP3*; F) CpG site +117 of *TIMP3*.

Little is known about the association between molecular alterations in tendon samples and clinical characteristics. The DNA methylation frequency at the CpG sites -536, -454 and -400 of *MMP1* was directly correlated with the age of patients in the CC samples (p<0.05 for all comparisons; Fig 4A–4C), suggesting that *MMP1* methylation may contribute to its decreased expression and, therefore, to rotator cuff tears. However, the frequency of DNA methylation at some CpG sites of *TIMP3* was directly correlated with age (-234 and +117 CpG sites, Fig 4D and 4F), whereas at another site, an inverse correlation was observed (CpG + 27; Fig 4E). Advancing age progressively affects the risk and severity of several chronic diseases through epigenome modification, along with changes in DNA methylation, due to both random drift and variation within specific functional loci [38]. Further investigation is necessary to understand the influence of the aging process in *TIMP3* methylation.

**Fig 5:**
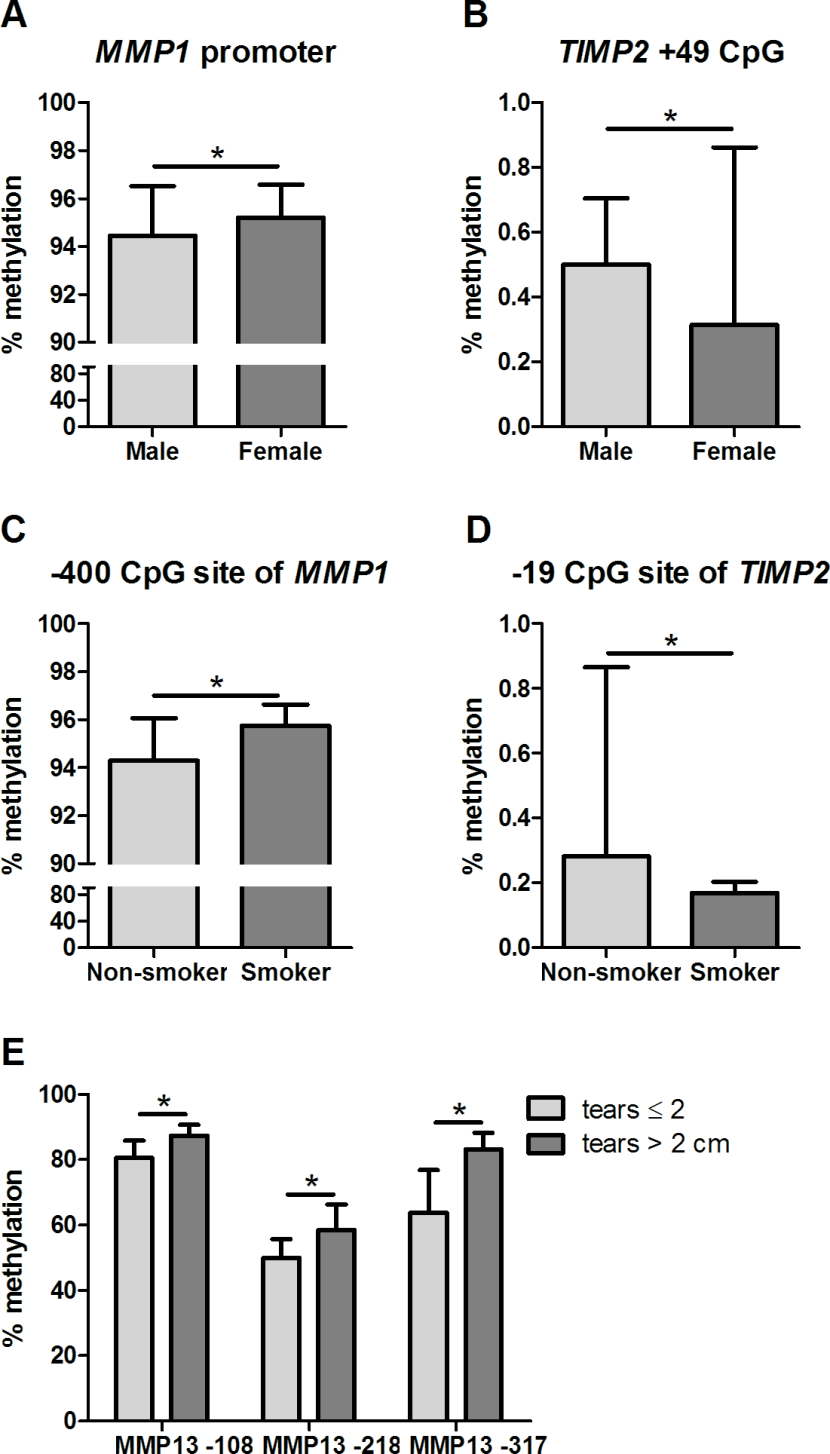
Clinical variations and DNA methylation in rotator cuff tears. A) Increased DNA methylation frequency at *MMP1* promoter in females; B) Reduced DNA methylation frequency at CpG site +49 of *TIMP2* in females; C) Increased DNA methylation frequency at CpG site -400 of *MMP1* in smokers; D) Reduced DNA methylation frequency at CpG site -19 of *TIMP2* in smokers; E) Increased DNA methylation frequency in CpG sites of *MMP13* in samples of patients with tears greater than 2 cm. *Significant difference between groups by Mann-Whitney test (p<0.05).

Rotator cuff tears occur more frequently in males than in females [39]. The prevalence of this disease in females can be partly explained by hormonal variation [40], which influences the tendon biology, affecting both the collagen and the ECM metabolism at structural and biochemical levels [41, 42]. The frequency of DNA methylation at the *MMP1* promoter was higher in females than in males (p = 0.043; Fig 5A). Moreover, the DNA methylation frequency at the CpG site +49 of *TIMP2* was lower in females than in males (p = 0.013; Fig 5B). Thus, female hormones might serve as an intrinsic factor that influences the transcriptional regulation of *MMPs* and *TIMP*s through alterations in DNA methylation.

Smoking is a known risk factor in rotator cuff tears [42] and contributes to failed tendon healing [43]. The DNA methylation was increased at the CpG site -400 of *MMP1* (p = 0.031; Fig 5C) and was decreased at -19 of *TIMP2* (p = 0.044; Fig 5D) in the CC samples of smokers compared with nonsmokers. Our findings support that smoking is an environmental factor that might alter the DNA methylation in tendon samples and therefore contribute to the failure of tendon healing process or directly to the tissue tears.

The DNA methylation at the CpG sites -108 (p = 0.017), -218 (p = 0.045), and -317 (p = 0.036) of *MMP13* (two of which correlated with the gene expression) was increased in the CC samples of patients with tears > 2 cm compared with patients with tears ≤ 2 cm (Fig 5E). In our study, no association was found between *MMP13* expression and tear size. However, the findings of Klatte-Schulz et al. [44] indicated that the *MMP13* expression was decreased in larger tendon tears. Therefore, the downregulation of *MMP13* through epigenetic mechanisms may have a role mainly in larger tears.

**Fig 6:**
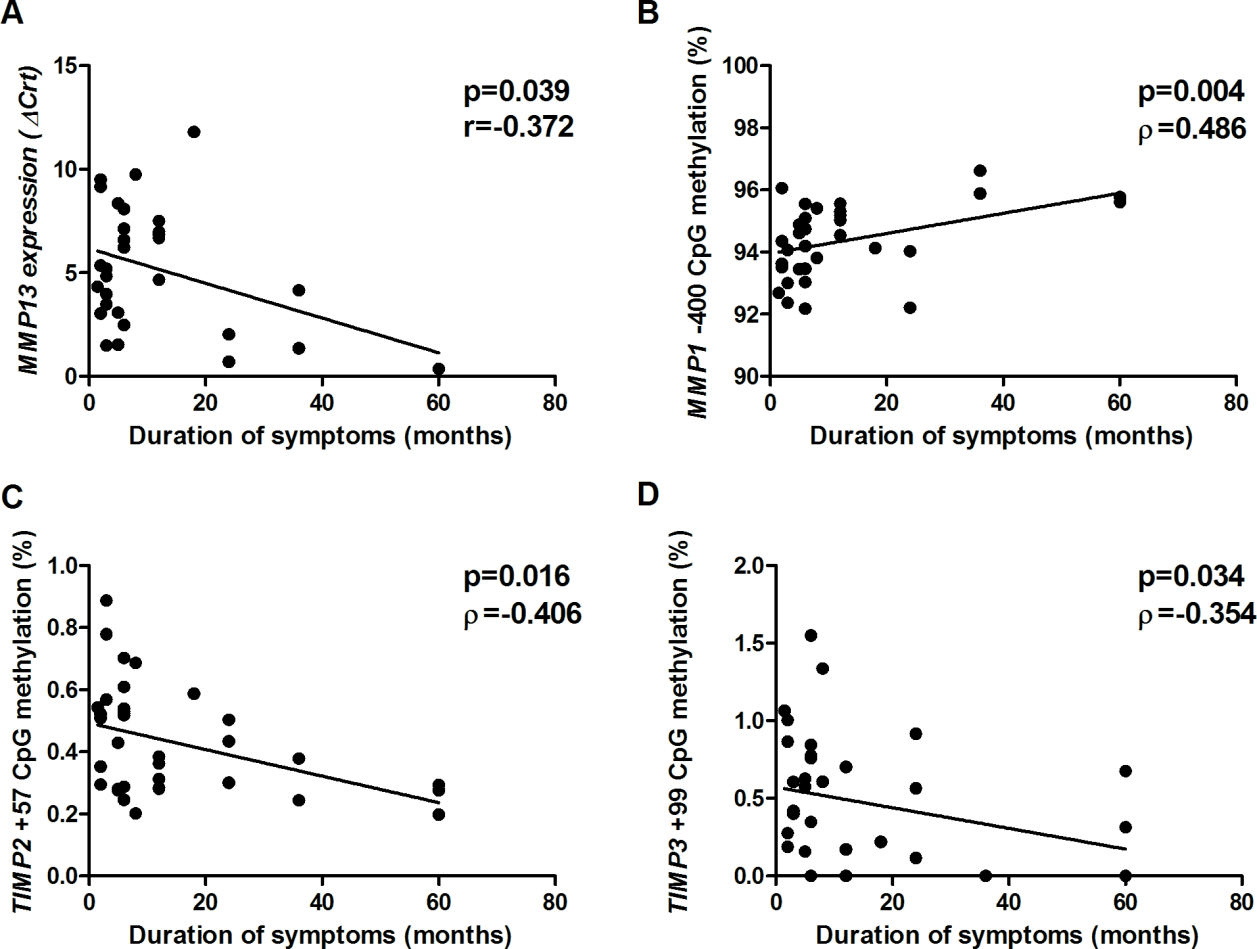
Correlation between duration of symptoms and molecular alterations in rotator cuff tears. A) *MMP13* expression; B) DNA methylation at the CpG site -400 of *MMP1*; C) DNA methylation at the CpG site +57 of *TIMP2*; D) DNA methylation at the CpG site +99 of *TIMP3*. A lower delta cycle threshold value (ΔCrt) indicates higher gene expression.

Longer duration of symptoms also influences the gene expression and methylation in tendons (Fig 6). Longer duration of symptoms was associated increased methylation at the CpG site -400 of *MMP1* (Fig 6B) and decreased methylation at +57 of *TIMP2* and at +99 of *TIMP3* (Fig 6C and 6D). These findings suggest that during the disease progression, the DNA methylation pattern of some *MMPs* and *TIMPs* is continuously altered. Conversely, increased expression of *MMP13* was correlated with duration of symptoms (Fig 6A). This finding may contribute to the heterogeneity in ruptured tendon samples and to the increased *MMP13* expression observed in CC compared with AC samples, as described above.

### Alterations in *MMP* and *TIMP* lead to modifications in the ECM structure

**Fig 7:**
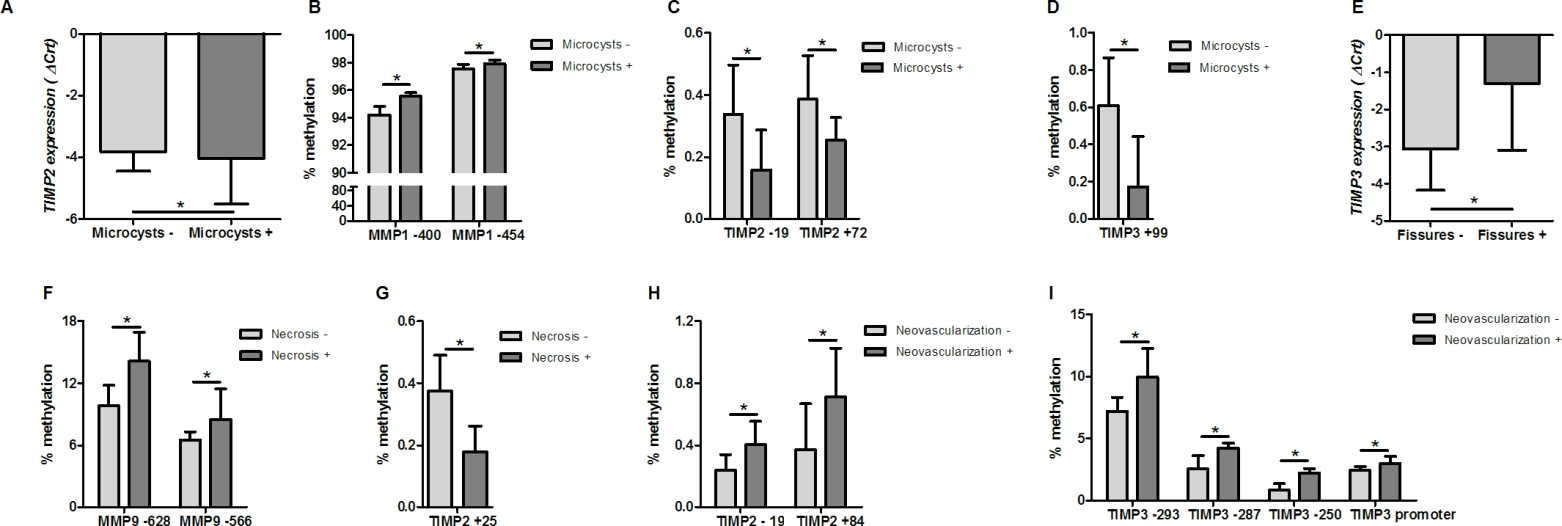
Associations between histological and molecular alterations in rotator cuff tears. A) Increased *TIMP2* expression in samples with microcysts; B) Increased *MMP1* methylation in samples with microcysts; C) Reduced *TIMP2* methylation in samples with microcysts; D) Reduced *TIMP3* methylation in samples with microcysts; E) Reduced *TIMP3* expression in samples with fissures; F) Increased *MMP9* methylation in samples with degenerative/necrotic changes; G) Reduced *TIMP2* methylation in samples with degenerative/necrotic changes; H) Increased *TIMP2* methylation in samples with neovascularization; I) Increased *TIMP3* methylation in samples with neovascularization. A lower delta cycle threshold value (ΔCrt) indicates higher gene expression. *Significant difference between groups (p<0.05) by t-test (gene expression data) or Mann-Whitney test (DNA methylation data).

Regarding the histologic characteristics, samples with increased *TIMP2* expression (p = 0.015; Fig 7A) and decreased methylation at two CpG sites (CpG -19: p = 0.003; CpG +72: p = 0.023; Fig 7C), as well as at one CpG site of *TIMP3* (CpG +99: p = 0.030; Fig 7D), presented microcysts more frequently, thus supporting the hypothesis that these MMP inhibitors may play a role in the degenerative process. Conversely, the methylation of *MMP1* was also associated with this histologic finding (CpG -400: p = 0.006; CpG -454: p = 0.049; Fig 7B), which agrees with our hypothesis that decreased *MMP1* expression may contribute to rotator cuff tears.

Tissue samples with necrosis showed increased DNA methylation at the CpG sites -628 and -566 of *MMP9* (p = 0.035 and p = 0.009, respectively; Fig 7F) and decreased methylation at +25 of *TIMP2* (p = 0.037; Fig 7G). Moreover, a significant increase in necrosis frequency was observed in patients with longer duration of symptoms [median duration (IQR): 24 (36) vs 6 (10.5) months; p = 0.032). Thus, the DNA methylation pattern of some *MMPs* and *TIMPs* is continuously altered during the disease progression with an impact on the tissue structure. Conversely, increased expression of *MMP13* was correlated with duration of symptoms.

Neovascularization is a common finding in rotator cuff disease [11, 45]. Although the key drivers of neovessel formation in chronic tendon injuries remain the subject of ongoing research, there is strong evidence that the process of neovascularization involves elements of the inflammatory response [45]. Neovascularization was associated with the increased DNA methylation at CpG sites of *TIMP2* (CpG -19: p = 0.047; CpG +84: p = 0.030; Fig 7H) and *TIMP3* (CpG -293: p = 0.006; CpG -287: p = 0.046; CpG -250: p = 0.006; CpG +108 p = 0.033; mean: p = 0.040; Fig 7I). The increased DNA methylation of *TIMPs* may favor the decreased expression of these genes following the increased protein expression of MMPs in some of the rotator cuff tear samples. The increased expression of MMPs is important for the inflammatory process. Multiple MMPs have been found to play key roles in inflammation, specifically in the migration of leukocytes through connective tissue, as well as in tissue destruction, remodeling, and angiogenesis [46].

Additionally, as described above, our results suggest that there is an overall attempt to repair the tissue structure at the edge of the ruptured supraspinatus, as indicated by the observed increased expression of ECM genes [3, 7–9], decreased expression of MMPs, and increased expression of TIMPs. However, some tissue samples also presented necrosis processes, and these samples more frequently showed increased *MMP9* and decreased *TIMP2* methylation. Furthermore, decreased *TIMP3* expression was found in samples with fissures, which may favor the degradation of ECM proteins by MMPs. No samples presented fissures or necrosis combined with neovascularization. Although the results are still preliminary, our study showed that a rotator cuff tear is a heterogeneous disease and that the edge of the torn supraspinatus presents different histologic and molecular alterations.

## Conclusions

Dysregulated expression and DNA methylation of *MMPs* and *TIMPs* occur across the rotator cuff tendon samples of patients with supraspinatus tears. Thus, we originally described that these alterations can be “spread” to non-rupture human tendons (by clinical and imaging analyses) highlighting the effect of a tendon tear in the shoulder joint complex, which should be considered during the patient management since it can increase the risk of other tendon lesions. It is also worth noting that the understanding that epigenetic modifications contribute to tendon tears is extremely relevant since they represent changes that are possible to be modified by external factors such as life habits, medications and physical activity. In fact, we demonstrated that dysregulated expression and DNA methylation of *MMPs* and *TIMPs* are influenced, at least in part, by clinical characteristics, such as age at surgery, sex, smoking habit, tear size, and duration of symptoms. Our results indicate that these alterations may lead to modifications in the ECM structure, which contribute to the presence of microcysts, fissures, necrosis, and neovascularization in tendons and thus may be involved in the tendon healing process. Therefore, *MMPs* and *TIMPs* are regulated by epigenetic modifications and may play a role in rotator cuff tears.

## Supporting information

S1 Fig Biopsy sites in a right shoulder specimen from cadaver without rotator cuff tear. Sagital plane indicating where the tissue samples representative of the three sectors of the rotator cuff were collected. AC (anterior cuff), CC (Central cuff) and PC (posterior cuff). This photo is only illustrative and this specimen was not used in the present study.
(JPG)

S2 Fig Expression of miR-29 members in tendon samples of individuals with and without rotator cuff tears. A) *miR-29a-3p*; B) *miR-29a-5p*; C) *miR-29b-3p*; D) *miR-29b-5p*. A lower delta cycle threshold value (ΔCrt) indicates higher gene expression. CC: central cuff (the torn supraspinatus edge); PC: posterior cuff, which represents a supraspinatus tendon sample without macroscopic alteration with native footprint insertion; AC: anterior cuff (subscapular tendon); EC: external control representing tendon samples of patients without rotator cuff tears.
(TIF)

S3 Fig Correlation between gene expression and DNA methylation in tendon samples. A lower delta cycle threshold value (ΔCrt) indicates higher gene expression. Therefore, a positive rho (ρ) may be inferred as an inversely correlation.
(TIF)

S1 File Targeted bisulfite amplicon sequencing. (DOCX)

S2 File Clinical and histological variables, gene expression and DNA methylation of each rotator cuff tear patient and control. (XLSX)

S1 Table Summary of the reference genes and target gene assays. (DOCX)

S2 Table Primer sequences (5’-3’) for methylation analysis. (DOCX)
