# Supplementary figures and images for: Epigenetic regulation of metalloproteinases and their inhibitors in rotator cuff tears
Source: PLoS One. 2017 Sep 13;12(9):e0184141. doi: 10.1371/journal.pone.0184141 (PMC5597200; doi:10.1371/journal.pone.0184141)

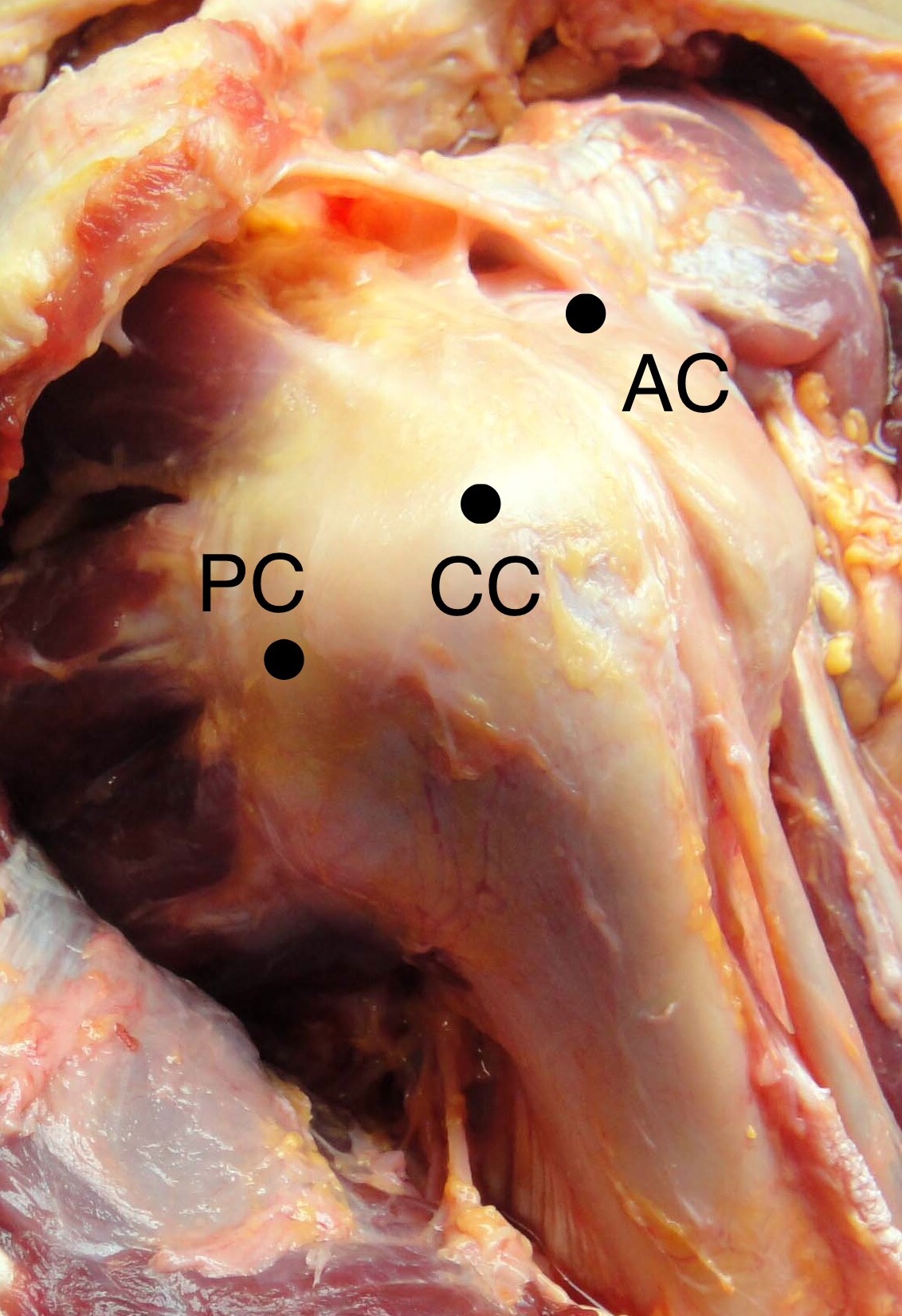

Supplement: S1 Fig — Sagital plane indicating where the tissue samples representative of the three sectors of the rotator cuff were collected. AC (anterior cuff), CC (Central cuff) and PC (posterior cuff). This photo is only illustrative and this specimen was not used in the present study. (JPG) [file pone.0184141.s001.jpg]

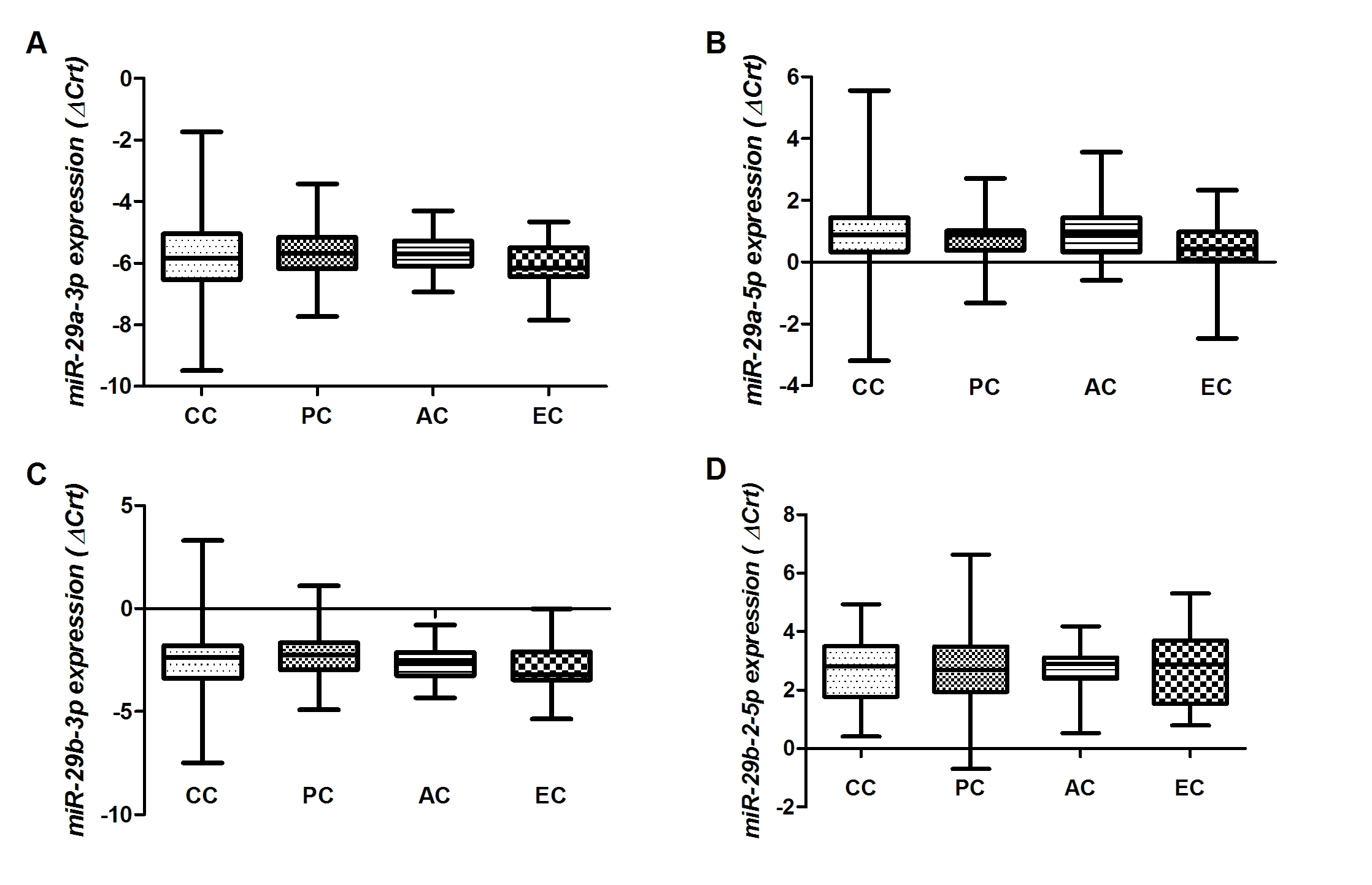

Supplement: S2 Fig — A) miR-29a-3p; B) miR-29a-5p; C) miR-29b-3p; D) miR-29b-5p. A lower delta cycle threshold value (ΔCrt) indicates higher gene expression. CC: central cuff (the torn supraspinatus edge); PC: posterior cuff, which represents a supraspinatus tendon sample without macroscopic alteration with native footprint insertion; AC: anterior cuff (subscapular tendon); EC: external control representing tendon samples of patients without rotator cuff tears. (TIF) [file pone.0184141.s002.tif]

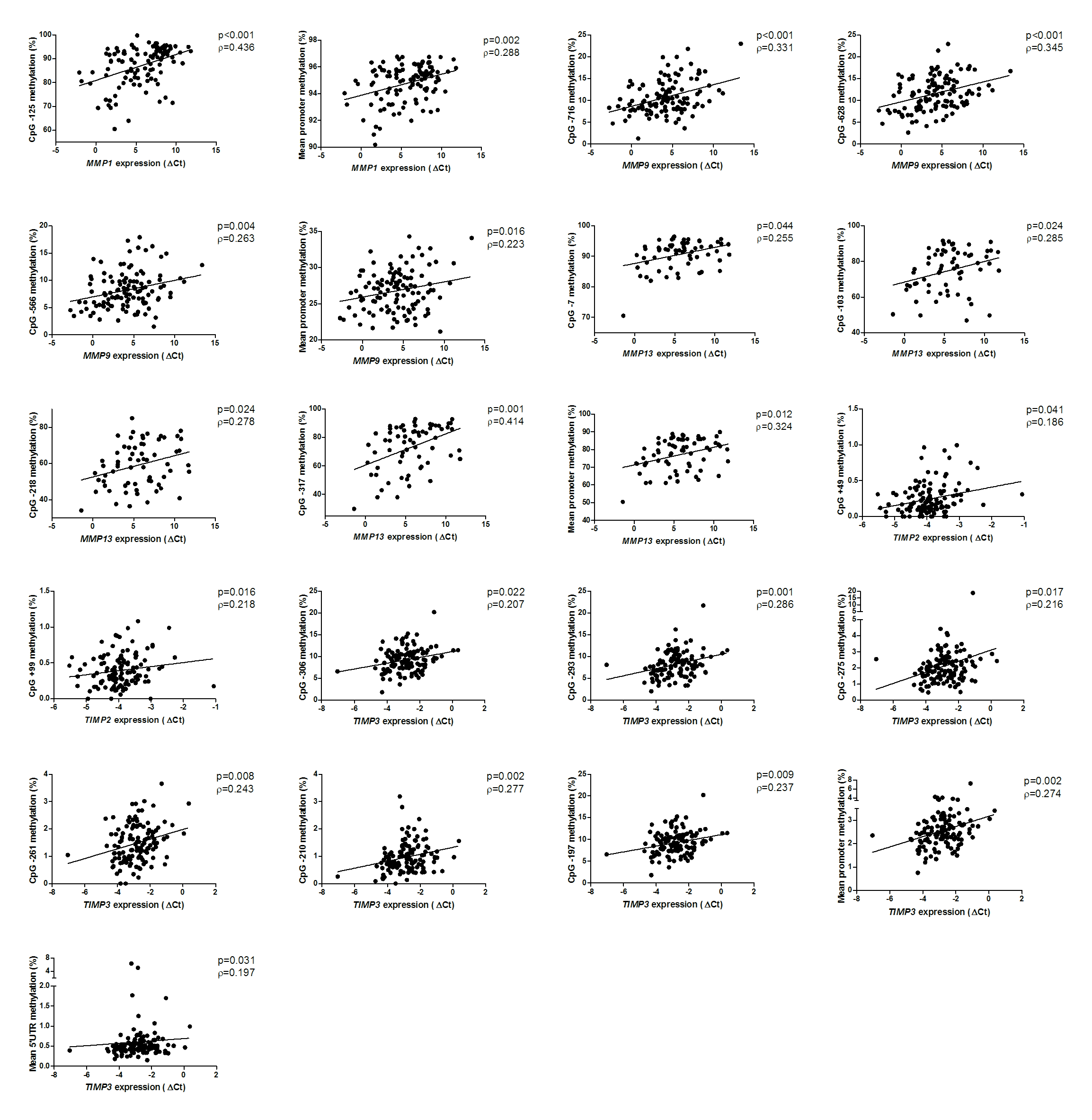

Supplement: S3 Fig — A lower delta cycle threshold value (ΔCrt) indicates higher gene expression. Therefore, a positive rho (ρ) may be inferred as an inversely correlation. (TIF) [file pone.0184141.s003.tif]
