# Supplementary material for: Epigenetic regulation of metalloproteinases and their inhibitors in rotator cuff tears
Source: PLoS One. 2017 Sep 13;12(9):e0184141. doi: 10.1371/journal.pone.0184141 (PMC5597200; doi:10.1371/journal.pone.0184141)
Supplement: S1 File — (DOCX) [file pone.0184141.s004.docx]

**Supporting information**

**S1 File.** **Targeted bisulfite amplicon sequencing**

After bisulfite treatment, PCR reactions were carried out by using 0.2 mM dNTPs, 2 mM MgSO4, 0.5 μM primers, 1.25 U Platinum^®^ Taq DNA Polymerase High Fidelity (Invitrogen, USA), and 20 ng bisulfite-modified DNA. After initial denaturation at 94 ºC for 5 min, 40 cycles at 94 ºC for 30 s, at the annealing temperature (S2 Table) for 30 s, and at 72 ºC for 30 s were carried out, followed by a final extension at 72 ºC for 7 min.

Before sequencing, the PCR products were visualized by using 1.5% agarose gel electrophoresis. As control of the bisulfite conversion and sequencing reaction, commercial human methylated and non-methylated DNA samples were also evaluated (Zymo Research, USA).

The sample generated two equimolar pools of amplicons, which were purified by using Agencourt AMPure XP beads (bead/DNA ratio, 1.8:1; Beckman Coulter, USA). About 10-50 ng of DNA was used as the input for library preparation. Libraries for next-generation sequencing were prepared by using the GeneRead DNA Library L Core kit (Qiagen, USA) and the Ion Xpress^TM^ Barcode Adapters kit (Thermo Fisher, USA). The final library was purified with the use of the Size Selection kit (Qiagen, USA) and Agencourt AMPure XP beads (bead/DNA ratio, 1.5:1; Beckman Coulter, USA). The libraries were amplified (8 cycles) by using the GeneRead DNA Library L Amp kit (Qiagen, USA).

Quality control for the library was carried out by measuring the DNA concentration with the Qubit dsDNA HS assay (Life Technologies, USA) on a QuBit 2.0 fluorometer (Life Technologies, USA).

Next, the multiplexed barcode libraries were enriched by clonal amplification by applying emulsion PCR on Ion Sphere particles (Ion PGM^TM^ Hi-Q^TM^ OT2 kit; Thermo Fisher, USA) in the Ion OneTouch 2 system (Thermo Fisher, USA) and then in the Ion OneTouch ES module (Thermo Fisher, USA). Sequencing was done on a PGM sequencer (Ion Torrent; Thermo Fisher, USA) with the use of the Ion PGM^TM^ Hi-Q^TM^ Sequencing kit (Thermo Fisher, USA) and the Ion 318 Chip kit v2 (Thermo Fisher, USA).

After sequencing, the raw signal data were analyzed by using the Torrent Suite software version 3.0 (Thermo Fisher, USA). The standard pipeline included signaling processing, base calling, quality score assignment, adapter trimming, mapping to GRCH37/hg19 reference, and detection of mapping quality.
