# Supplementary material for: Epigenetic regulation of metalloproteinases and their inhibitors in rotator cuff tears
Source: PLoS One. 2017 Sep 13;12(9):e0184141. doi: 10.1371/journal.pone.0184141 (PMC5597200; doi:10.1371/journal.pone.0184141)
Supplement: S1 Table — (DOCX) [file pone.0184141.s006.docx]

**Supporting information**

**S1 Table.** Summary of the reference genes and target genes assays.

| **Gene symbol** | **Assay**^a^ |
| --- | --- |
| *MMP1* | Hs00899658_m1 |
| *MMP2* | Hs01548727_m1 |
| *MMP3* | Hs00968305_m1 |
| *MMP9* | Hs00234579_m1 |
| *MMP13* | Hs00233992_m1 |
| *MMP14* | Hs01037003_g1 |
| *TIMP1* | Hs00171558_m1 |
| *TIMP2* | Hs00234278_m1 |
| *TIMP3* | Hs00165949_m1 |
| *ACTB*^b^ | Hs01060665_g1 |
| *TBP*^b^ | Hs00427620_m1 |
| *HPRT1*^b^ | Hs02800695_m1 |
| *miR-29a-3p* | 002112 |
| *miR-29a-5p* | 002447 |
| *miR-29b-3p* | 000413 |
| *miR-29b-5p* | 002166 |
| *miR-29c-3p* | 000587 |
| *miR-29-5p* | 001818 |
| *U6*^c^ | 001973 |

^a^TaqMan probes were purchased as assays-on-demand products for gene expression (Life Technologies, USA); ^b^Reference genes for target mRNA expression normalization; ^c^Reference genes for target miRNA expression normalization.
