# Supplementary material for: Epigenetic regulation of metalloproteinases and their inhibitors in rotator cuff tears
Source: PLoS One. 2017 Sep 13;12(9):e0184141. doi: 10.1371/journal.pone.0184141 (PMC5597200; doi:10.1371/journal.pone.0184141)
Supplement: S2 Table — (DOCX) [file pone.0184141.s007.docx]

**Supporting information**

**S2 Table.** Primer sequences (5’-3’) for methylation analysis.

| Gene | Amplicon | Sense | Antisense | Anneling temperature (^o^C) | Product size (bp) | CpG sites analyzed |
| --- | --- | --- | --- | --- | --- | --- |
| *MMP1* | 1 | 5' ATGTTGTTTAGGTTGATTTTGA 3' | 5' AAATTCCCTTCTACCTTTATTAAAC 3' | 53 | 177 | 2^a^ |
|  | 2 | 5' TTTTGAGTAAAGATTAAGGGAAGTTATG 3' | 5' CCTTACTCCCAAAACAAAAAATAAA 3' | 58 | 203 | 5^a^ |
|  | 3 | 5' TGAATTGGAGAAAATTATTGTT 3' | 5' CCAACTAAAAAACTCCCTCTAT 3' | 52 | 244 | 1 |
| *MMP9* | 1 | 5' GAATTTTGGGTTTTGGTTTTAGTAAT 3' | 5' ACAAACATAACTTTACTCTCTTCCTTCA 3' | 59 | 231 | 4 |
|  | 2 | 5' TGTTTTTTAGAGGTTGTTATTGTT 3' | 5' ACCTTCTTTAACTCAACTTCCTC 3' | 57 | 158 | 2 |
|  | 3 | 5' GATGGGGGATTTTTTTAGTTTT 3' | 5' AAACTTACACCACCTCCTCCT 3' | 57 | 181 | 3^b^ |
| *MMP13* | 1 | 5' AATTAGTATTAAGTTTTTTTTTATGGAAGT 3' | 5' TTCAACAAAATCTCAAAACCCATCTAA 3' | 57 | 216 | 3 |
|  | 2 | 5' ATGGGTTTTGAGATTTTG 3' | 5' ACCCCTAAATACATCTTAAATA 3' | 52 | 246 | 4 |
| *TIMP2* | 1 | 5' GAGYGTTTAGAGTTTGTATTGG 3' | 5' TTTCTCTCCTCTTTATCTCRAA 3' | 54 | 233 | 28^c^ |
| *TIMP3* | 1 | 5' AGAGATATTTAGTGGTTTAGGTGGG 3' | 5' TTCAAATCCTTATAAAAAATAATACC 3' | 53 | 161 | 15 |
|  | 2 | 5' AGAGYGGGTAGTAGGTAGG 3' | 5' TACRAAACAACCTTAACCTCC 3' | 60 | 191 | 21^c^ |

^a^1 CpG is common between the amplicon #1 and #2 of *MMP1*. A total of 7 CpG in *MMP1* were evaluated. ^b^2 CpGs of amplicon #3 of *MMP9* had low covered during the sequencing and were not considered for statistical analysis. A total of 7 CpG sites in *MMP9* were evaluated. ^c^CpG sites at degenerate primers were not included in the counting.
